# TEPIC 2—an extended framework for transcription factor binding prediction and integrative epigenomic analysis

**DOI:** 10.1093/bioinformatics/bty856

**Published:** 2018-10-09

**Authors:** Florian Schmidt, Fabian Kern, Peter Ebert, Nina Baumgarten, Marcel H Schulz

**Affiliations:** 1 High throughput Genomics and Systems Biology, Cluster of Excellence on Multimodal Computing and Interaction, Saarland Informatics Campus, Saarbrücken, Germany; 2 Computational Biology and Applied Algorithmics, Max Planck Institute for Informatics, Saarland Informatics Campus, Saarbrücken, Germany; 3 Graduate School of Computer Science, Saarland Informatics Campus, Saarbrücken, Germany; 4 Chair for Clinical Bioinformatics, Saarland Informatics Campus, Saarbrücken, Germany; 5 Institute for Cardiovascular Regeneration, Goethe University, Partner site Rhein-Main, Frankfurt am Main, Germany; 6 German Center for Cardiovascular Research, Partner site Rhein-Main, Frankfurt am Main, Germany

## Abstract

**Summary:**

Prediction of transcription factor (TF) binding from epigenetics data and integrative analysis thereof are challenging. Here, we present TEPIC 2 a framework allowing for fast, accurate and versatile prediction, and analysis of TF binding from epigenetics data: it supports 30 species with binding motifs, computes TF gene and scores up to two orders of magnitude faster than before due to improved implementation, and offers easy-to-use machine learning pipelines for integrated analysis of TF binding predictions with gene expression data allowing the identification of important TFs.

**Availability and implementation:**

TEPIC is implemented in C++, R, and Python. It is freely available at https://github.com/SchulzLab/TEPIC and can be used on Linux based systems.

**Supplementary information:**

Supplementary data are available at *Bioinformatics* online.

## 1 Introduction

Transcription Factors (TFs) are key players of transcriptional regulation. Prediction of TF binding is essential to gain a deeper understanding of their function. While experimental identification of TF binding is possible through laborious and expensive ChIP-seq assays, several computational approaches have been proposed to identify TF binding sites (TFBSs) (Jayaram *et al.*, 2016). These predictions have been successfully augmented using epigenetics data (Cuellar-Partida *et al.*, 2012; Pique-Regi *et al.*, 2011; Sherwood *et al.*, 2014). As delineated in Supplementary Section 1, *TEPIC 2* builds upon and extends the functionality of existing TFBS prediction tools. Among other features, *TEPIC 2* allows the direct aggregation of TFBS predictions on the gene level and uses these scores to gain novel insights on cell type specific functions of TFs via several machine learning analysis. This is a unique feature not supported by competitive TFBS prediction approaches (Supplementary Tables S1 and S2). Compared to its predecessor, *TEPIC 2* has substantially lower runtime, contains an extended set of TF motifs, offers various means for downstream machine learning analyses as easy-to-use pipelines, and adds new functionalities to compute TF gene scores.

## 2 Features

The core functionalities of *TEPIC 2* are to predict TFBS in user provided regions and to aggregate them to TF gene scores. The TF gene score computation has been modified to compute statistical features such as region length, region count, and the signal of an epigenetic assay within the considered regions. *TEPIC 2* can compute a binary binding assessment, i.e. a TF binds or does not bind, based on *p*-values obtained using a set of background regions of similar characteristics as the input set. This feature complements the continuous TF affinity values of TRAP, which are not suitable for all downstream applications (Supplementary Section 4).

Additionally, the aforementioned TF gene scores can be used in several integrative analysis workflows (Supplementary Section 7, 8 and 9). *INVOKE* refers to a sparse linear regression model to reveal key TFs potentially regulating transcription. It highlighted several known tissue-specific regulators in liver hepatocytes and CD4+ T cells (Schmidt *et al.*, 2017) and is also available as a web-server (Kehl *et al.*, 2017). Besides, *TEPIC 2* includes a sparse logistic regression classifier to infer TFs related to gene expression changes between samples (*DYNAMITE*). *DYNAMITE* has been successfully applied to discover regulators of CD4+ T cell differentiation (Durek *et al.*, 2016). Recently, we combined TEPIC with DREM (Schulz *et al.*, 2012) to uncover master regulatory TFs from paired time-series expression and epigenomics data (*EPIC-DREM*), which was used to analyze mesenchymal stem-cell differentiation of osteoblasts and adipocytes (Gerard *et al.*, 2018).

Furthermore, we considerably extended the set of TF motifs readily available in *TEPIC 2*. Now, this resource contains 30 species-specific and six taxonomy-specific sets from JASPAR (Mathelier *et al.*, 2016), as well as aggregated sets for humans, mice and vertebrates containing 561, 380 and 690 TF motifs (Supplementary Section 3). To streamline the training and interpretation of statistical models (Supplementary Fig. S1), we provide clustered versions of the merged TF motif files, representing families of binding motifs with high similarity (Pape *et al.*, 2008).

## 3 Implementation

**Fig. 1.**
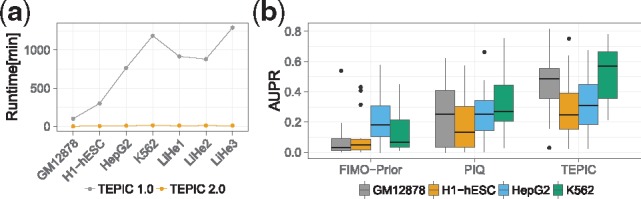
(**a**) Runtime comparison of *TEPIC* to *TEPIC 2* using a subset of 458 human TFs. While the original implementation ran up to 1300 minutes to compute TFBS, *TEPIC 2* is able to compute TF affinities for peaks in the vicinity of genes in at most 15 minutes. We used four cell line samples and three primary human hepatocyte samples (LiHe1–3) to conduct the runtime experiments. (**b**) We compared *TEPIC* TF affinities computed in footprints called with HINT-BC (Gusmao *et al.*, 2016) in four different cell-lines in terms of AUPR against PIQ (Sherwood *et al.*, 2014) and an extension of the widely used method Fimo, called Fimo-Prior (Cuellar-Partida *et al.*, 2012). Notably, TF affinities computed with TEPIC outperform both PIQ and Fimo-Prior

*TEPIC 2* uses a parallelized C++ implementation of TRAP (Roider *et al.*, 2007) that is considerably faster than the previous *R* implementation. Runtime was further reduced by using more efficient search algorithms and by enabling pre-filtered analyses of samples in minutes (Fig. 1a, Supplementary Table S5, Supplementary Fig. S2 and Supplementary Section 5). We evaluated the accuracy of TFBS predictions from *TEPIC 2* using TF footprints called with HINT-BC (Gusmao *et al.*, 2016) on ENCODE data (The ENCODE Project Consortium, 2012). In comparison to established tools for TFBS prediction using epigenomics data (Cuellar-Partida *et al.*, 2012; Sherwood *et al.*, 2014), *TEPIC* performs favorably in terms of area under the precision recall curve (AUPR) (Fig. 1b, Supplementary Fig. S3 and Supplementary Section 6). Details on samples used are provided in Supplementary Section 2. The machine learning pipelines included in *TEPIC 2* are implemented in *R*. Both workflows deliver results that are easy to interpret, also for non-expert users, due to automated figure generation and extensive documentation. As input, the pipelines require standard file formats, e.g. *bed* files for candidate TFBS and tab delimited *txt* files containing gene expression data. *TEPICs* full functionality is brought to the user via *start-to-finish* pipelines, which are automatically installed with *TEPIC 2*.

## 4 Conclusion

TEPIC 2 is a fast and easy-to-use tool for TFBS prediction combined with integrative analysis capabilities for gene expression and epigenomic data. TFBS prediction and downstream machine learning pipelines for various analysis settings allow a deep, seamless exploration of epigenomic datasets supporting data driven hypothesis generation about the role of individual TFs in complex regulatory landscapes.

## Supplementary Material

Supplementary Material
