# Supplementary material for: TEPIC 2—an extended framework for transcription factor binding prediction and integrative epigenomic analysis
Source: Bioinformatics. 2018 Oct 9;35(9):1608–9. doi: 10.1093/bioinformatics/bty856 (PMC6499243; doi:10.1093/bioinformatics/bty856)
Supplement: Supplementary Material [file bty856_supplementary_material.pdf]

Supplementary Material

TEPIC2 – An extended framework for transcription factor binding prediction and integrative epigenomic analysis

Florian Schmidt, Fabian Kern, Peter Ebert, Nina Baumgarten, Marcel H. Schulz

Overview 2
1 Methods for transcription factor binding site prediction and integrative analysis 3
2 Data & Preprocessing 7
3 Position specific energy matrices (PSEMs) 11
4 Score computation in TEPIC 13
5 Runtime evaluation of TEPIC 15
6 Assessment of TFBS predictions using ChIP-seq data 18
7 Inferring essential regulators using linear regression (INVOKE) 22
7.1 Motivation 22
7.2 Description of the pipeline 22
7.3 Example 25
8 Determining regulator of differential gene expression (DYNAMITE) 27
8.1 Motivation 27
8.2 Description of the pipeline 27
8.3 Example 29
9 Identification of important regulators from time series epigenomics and expression data (EPIC-DREM) 31
9.1 Motivation 31
9.2 Description 31
10 References 33

Overview

TEPIC2 is a versatile tool for the analysis of transcription factor (TF) binding and offers several machine learning approaches for integrative analysis of predicted transcription factor binding sites (TFBS) and gene expression data.

Briefly, TEPIC2 offers:

- Annotation of user-defined regions with TF affinities using TRAP and a variety of provided TF motifs obtained,
- Aggregation of TF affinities to TF gene scores,
- Computation of statistical scores such as peak-length, peak-count and peak-signal per gene,
- Discretization of continuous TF affinities using a background distribution into a binary measure for TF binding,
- Linear regression analysis to infer key transcriptional regulators within one sample (INVOKE),
- Logistic regression classifier to suggest key transcriptional regulators between samples (DYNAMITE),
- Generate input for DREM to infer key TFs from temporal epigenomic and gene expression data (EPIC-DREM).

An overview on the possible analyses is shown in Figure 1. The Supplementary material is based on text from the TEPIC2 ReadMe file, the online documentation included in the repository, the original TEPIC publication [0], its supplement, and on the supplement of [14].

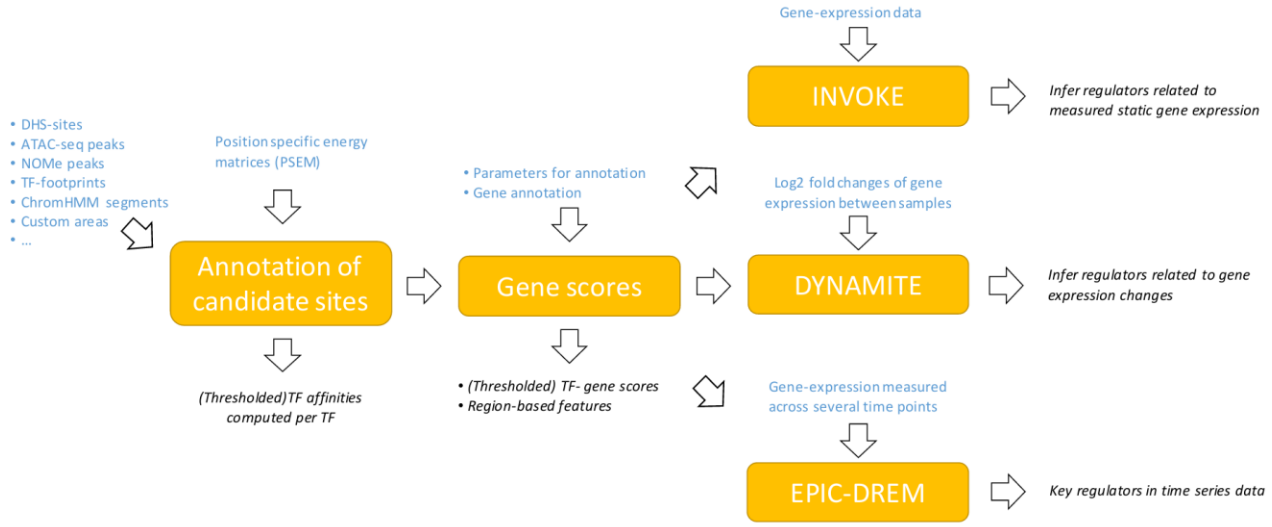

Supplementary Figure1: Overview of the basic workflow supported by TEPIC2. The input to the submodules is shown in blue, whereas the output is shown in black italic font.

1 Methods for transcription factor binding site prediction and integrative analysis

This chapter is partly based on a review article on predicting transcription factor binding [46] and on our original TEPIC publication [0].

As described in [46] there are several methods to predict transcription factor binding sites (TFBSs) using position specific weight matrices (PWMs) for individual TFs, e.g. *Matrix-Scan* [47], *Clover* [48], *Fimo* [26], and *PoSSuMsearch* [49].

Matrix-Scan computes a log ratio score per sequence comparing the probability of motif hit in S against a background model [47]. Similarly, *Clover* computes a log-ratio score and in addition computes a p-value to assess the scores significance using, e.g. permutation experiments and also corrects for multiple testing [48]. The widely used method *Fimo* computes a log-likelihood ratio score for each distinct sequence position against a zero-order background model and computes a p-value per site using dynamic programming and supports correcting for multiple hypothesis testing too [26]. *PoSSuMsearch* builds a suffix array of the considered sequence to speed up search time. Also, it offers a dynamic programming approach to come up with matrix specific thresholds based on a user defined E- or p-value [51]. The aforementioned tools have been systematically compared in [46], where it turned out that *Fimo* is outperforming the competing approaches. All evaluated methods are available as a local software installation, allowing them to be applied large-scale. Additionally, some methods can also be used as a webserver, for instance. *Fimo* [26]. While the list of methods considered in [46] is extensive, it is still not exhaustive, e.g. the method *TRAP* [13] used in *TEPIC* is not considered. This is underlining that an exhaustive listing and comparison of all TFBS predictions methods is hardly feasible, due to the large number of available methods. An overview on purely sequence based methods mentioned above is provided in Supplementary Table 1.

Method	Available	Supports parallel execution	Hit-based	Affinity-Based	Maintained set of PWMs	TF gene score computation	Supports linear analysis	Supports differential analysis
**Matrix-Scan**	Registration required	NA	Yes	No	NA	No	No	No
**Clover**	Yes	No	Yes	No	No	No	No	No
**Fimo**	Yes	No	Yes	No	No	No	No	No
**PoSSuMsearch**	Yes	Yes	Yes	No	No	No	No	No
**TRAP**	Yes	No	No	Yes	No	No	No	No
**DEEP-Bind**	Yes	Yes	<NA>	<NA>	No	No	No	No

Supplementary Table 1: Characteristics and Features of various, purely sequence based, tools for TFBS prediction and analysis. An entry of <NA> indicates that this particular characteristic is not applicable to a certain method.

Applying such predictions methods as the ones mentioned above genome-wide generates many false-positive hits compared to TF ChIP-seq experiments. Because it was observed that TFs usually bind to regions of open-chromatin [20, 25, 36], the false-positive rate could be reduced by the inclusion of epigenetics data. There are two general classes of methods using epigenetics data to improve TFBS predictions: (1) site-centric methods and (2) segmentation-based methods.

Site-centric methods require the identification of putative TFBSs in the entire genomic search space. According to one or multiple epigenetic signatures, the putative TFBSs are either classified as bound or unbound in a post processing step. This strategy has been pursued in many applications: For example, in *Centipede*, not only chromatin accessibility, but also histone modifications, genome conservation and the distance of a putative binding site to the closest TSS are combined in a hierarchical mixture model [25]. This method has been simplified in [17], where an epigenetic prior is computed from DNaseI-seq

data, which is then combined with a motif score computed with *Fimo* [26]. In *PIQ* [18], TFBS are predicted with Bayesian inference considering both PWM scores and epigenetics data.

In addition to these unsupervised methods, also supervised methods have been proposed, for instance *MILLIPEDE* [27] and *BinDNase* [28]. These tools attempt to learn a TF specific signature of the epigenetic signal around putative TFBS. Specifically, they use a binned DNaseI-seq signal around candidate TFBSs as features in a regression approach to identify actual TFBSs.

Segmentation-based approaches, on the other hand, narrow down the genomic search space beforehand, e.g. to DNase hypersensitive sites, TF footprints, or putative enhancer and promoter regions identified e.g. with *ChromHMM* [29]. This reduces the runtime compared to site-centric approaches.

Especially TF footprints have been found to be highly predictive for true TFBSs and various tools have been suggested to identify them from both DNaseI-seq and ATAC-seq data [8, 30]. Footprints are believed to be caused by TFs that are bound to DNA, thereby preventing the DNaseI-seq enzyme from cutting or the transposase from inserting. Various footprint-callers have been proposed in literature. They are based on sliding windows [31] or hidden Markov models (HMM) [8, 32, 33]. *DNase2TF* is using a binomial z-score to interpret the depletion of DNaseI-seq reads around putative footprints [33], while *Wellington* applies a binomial test to identify footprints [34]. Recent footprint calling methods, like *HINT-BC*, allow for correcting the cleavage bias of the enzymes used to assess chromatin accessibility [35].

The drawback of calling footprints is that in addition to the peak calling step, which is required to narrow down the search space, the actual footprint calling itself needs to be performed, too. Depending on the method, not only DNase1-seq or ATAC-seq but also Histone Modification ChIP-seq data is required to run these calls. Also, it is being argued in literature that some TFs do not generate footprints because their binding is subtle and unstable. Thus, they do not remain bound long enough at a distinct genomic location to generate a footprint [33].

Importantly, the peak calling step for epigenetic data, e.g. DNase1-seq or ATAC-seq is not trivial either. Several peak callers have been suggested to account for the unique characteristics of the respective assays [37].

Another downside of all aforementioned approaches for TF binding prediction is the usage of hit-based motif screening algorithms, such as *Fimo* [26]. These methods use a threshold to classify a genomic site as a putative TFBS or not. Low affinity binding sites may be lost in this classification. However it was shown that low-affinity binding is essential in biology [38, 39].

To circumvent all of the aforementioned limitations and to incorporate the advantages of segmentation based methods, i.e. favorable runtime, our *TEPIC* method considers segments, which can be TF footprints, DHS sites, ATAC-peaks, NOME-peaks and so on and annotates those regions with *TRAP* [13] and a set of user provided position weight matrices (see Sup. Section 4 and [13] for further details). *TRAP* circumvents the drawback of hit-based methods by quantifying TF binding using a biophysical model producing affinity values for each TF. *TRAP* affinities have been used before in various applications [40, 41, 42, 43]. Furthermore, *TEPIC* readily aggregates TF binding predictions to the level of individual genes, given a gene annotation file, e.g. from Gencode. As shown in Sup. Tab. 1, this is not a

standard feature among other tools, although per gene scores have been postulated in literature before [15, 59], but current software does not allow for their direct computation.

We acknowledge that recent efforts have been made to replace PWMs with more sophisticated models describing within motif dependencies, e.g. slim models [44]. However, without the availability of large scale open-source databases such as JASPAR providing these kind of motif descriptions, they are (a) hard to be obtained by end-users and (b) not available for all TFs, limiting the possible research applications.

Recent deep-learning approaches, for instance DEEP-Bind [45], try to learn the sequence specificities of TFs de novo from large data sets. These approaches require a lot of data, special hardware to be trained and are not as interpretable as a classical PWMs or the slim models. Therefore, these tools have not been applied to many TFs, also limiting their practical usage for hands-on research.

Supplementary Table 2 holds an overview on several approaches for TFBS prediction and analysis that utilize epigenetics data, illustrating the characteristics and features of the individual methods.

Method	Available	Supports parallel execution	Hit-based	Affinity-Based	Maintained set of PWMs	TF gene score computation	Supports linear analysis	Supports differential analysis
**Centipede**	Yes	No	Yes	No	No	No	No	No
**Fimo-Prior**	Yes	No	Yes	No	No	No	No	No
**PIQ**	Yes	Requires qsub	Yes	No	No	No	No	No
**Millipede**	Yes	NA	Yes	No	NA	No	No	No
**BinDNase**	No	NA	Yes	No	NA	No	No	No
**TEPIC2**	Yes	Yes	(Yes)	Yes	Yes	Yes	Yes	Yes
**RACER**	Yes	NA	NA	NA	NA	NA	Yes	No

Supplementary Table 2: Characteristics and features of various tools for TFBS prediction and analysis that utilize epigenetics data in addition to the sequence specificity of the TFs. An entry of <NA> indicates that this particular characteristic is not applicable to a certain method.

While predicting TFBS throughout the genome is already helpful to gain an understanding on potential functions of TFs, e.g. via enrichment analysis, combining TFBS with gene expression data allows to establish a relation between TFs and expression of their target genes. Such experiments have been previously conducted with TF ChIP-seq data [15, 51, 52], as well as epigenetics data and predicted TF binding sites [20, 21, 22]. Unfortunately, these do not offer stand-alone software to carry out TFBS predictions, aggregate those to a gene level and feed them into a linear regression model to prioritize candidate regulators, all be it [21] provide a virtual environment to enhance the reproducibility of their results. In 2014, a tool called *RACER* has been published using TF ChIP-seq and miRNA data to predict gene expression. However, this tool does not support predicting TF binding events and is specifically tailored for the application in [50]. Within TEPIC 2, we offer a pipeline that can be easily used to predict TF binding from epigenetics data, compute TF gene scores and utilize those to build a linear model predicting steady state gene-expression. Additionally, it is possible to run only individual steps of the pipeline which allows for example to use TF ChIP-seq data, instead of predicted TFBS. Further details are provided in Section 8.

In addition to the linear analysis presented above, it is also of interest to identify regulators associated to differentially expressed genes, e.g. between tissues or healthy and diseased samples. Originally, this problem has been mainly tackled using TF ChIP-seq data and specialized peak callers have been developed to determine differential TF ChIP-seq peak calls [53]. As shown in [54] differential TF ChIP-seq signals are correlated to differential gene-expression and can thus be used to suggest regulators potentially driving expression differences. Unfortunately [54] is not providing a software package to generate these associations. Another observation has been made in [55]. Here, it is shown that the epigenetic landscape differs between cell-types and leads to a distinct, predictable, binding behavior of

TFs. In light of that, specialized tools have been developed to call differential peaks from epigenetics data, e.g. histoneHMM [56], or MAnorm [57]. While these tools identify differential peaks, e.g. differential Histone Marks, they are not able to associate these regions to TFs and subsequently link them to differentially expressed genes. Our DYNAMITE approach included in TEPIC2 predicts TF binding from epigenetic data, e.g. differential peak calls or even standard peak calls and uses a log ratio score between TF affinities computed for two conditions to identify regulators linked to differential gene expression. Further details are provided in Section 9.

As exemplified in [58], considering time is essential for comparative epigenomic studies. One of the widely used tools to suggest master regulators in time-series experiments is DREM [24]. DREM comes with a default set of TF ChIP-seq data used for the analysis. In [16], we have shown that the predictions of DREM can be improved by applying DREM on TFBS predictions using temporal epigenomic data. To this end, we added an output format to TEPIC which can be readily used as input for DREM.

2 Data & Preprocessing

For this article, we used data from the German epigenome program (DEEP) [1] and from ENCDOE [2]. Supplementary Table 3 lists an overview on the official DEEP sample IDs as well as the ENCODE accession numbers. From ENCODE, we obtained quantified gene expression data as well as DNase1-seq BAM files.

BAM files of DEEP RNA-Seq reads were produced with TopHat 2.0.11 [3], with Bowtie 2.2.1 [4] and NCBI build 37.1 in *--library-type fr-firststrand* and *--b2-very-sensitive* setting. Gene expression has been quantified using Cufflinks version 2.0.2 [5], the hg19 reference genome and with the options *frag-bias-correct, multi-read-correct*, and *compatible-hits-norm* enabled.

DEEP DNase1-seq bam files were created according to the DEEP GAL v1 process (http://doi.org/10.17617/1.2W). Alignments were produced with BWA [6], sorted with samtools [7], and duplicated reads were marked with Picard tools (http://broadinstitute.github.io/picard).

DNase hypersensitive sites (DHS) have been called with JAMM using default parameters for both ENCODE and DEEP data. All peaks passing the JAMM filtering step have been used for further analysis.

TF footprints for GM12878, HepG2, H1-hESCs, and K562 have been called using HINT-BC [8] and are available online (http://costalab.org/publications-2/dh-hmm/).

TF ChIP-seq data was obtained from ENCODE for several TFs for K562, GM12878, HepG2, and H1-hESCs in narrow peak format. Supplementary Table 4 provides an overview.

**DEEP Sample ID**	**Sample ID used in this study**
01_HepG2_LiHG_Ct1	HepG2
41_Hf01_LiHe_Ct	LiHe1
41_Hf02_LiHe_Ct	LiHe2
41_Hf03_LiHe_Ct	LiHe3
**DEEP File ID**	**Data Type**
01_HepG2_LiHG_Ct1_mRNA_K_1.LXPv1.20150508_genes.fpkm_tracking	Quantified mRNA
01_HepG2_LiHG_Ct1_DNase_S_1.bwa.20140719.bam	Dnase-1 seq
41_Hf01_LiHe_Ct_mRNA_K_1.LXPv1.20150508_genes.fpkm_tracking	Quantified mRNA
41_Hf01_LiHe_Ct_DNase_S_1.bwa.20131216.bam	Dnase-1 seq
41_Hf01_LiHe_Ct_mRNA_K_1.LXPv1.20150508_genes.fpkm_tracking	Quantified mRNA
41_Hf02_LiHe_Ct_DNase_S_1.bwa.20131216.bam	Dnase-1 seq
41_Hf03_LiHe_Ct_mRNA_K_1.LXPv1.20150508_genes.fpkm_tracking	Quantified mRNA
41_Hf03_LiHe_Ct_DNase_S_1.bwa.20150120.bam	Dnase-1 seq
ENCFF000DYC	Quantified mRNA of K562
ENCFF000SVN	DNase -1 seq of K562
ENCFF000CZF	Quantified mRNA of GM12878
ENCFF000SKV	DNase -1 seq of GM12878
ENCFF000SKW	DNase -1 seq of GM12878
ENCFF000SKZ	DNase -1 seq of GM12878
ENCFF000SLB	DNase -1 seq of GM12878
ENCFF000SLD	DNase -1 seq of GM12878

ENCFF000DHQ	Quantified mRNA of H1-hESC
ENCFF000DHS	Quantified mRNA of H1-hESC
ENCFF000DHU	Quantified mRNA of H1-hESC
ENCFF000DHW	Quantified mRNA of H1-hESC
ENCFF000SOA	DNase-1 seq of H1-hESC
ENCFF000SOC	DNase-1 seq of H1-hESC

Supplementary Table 3: DEEP and ENCODE sample IDs of RNA-seq and DNase1-seq data.

**ENCODE Accession number**	**TF ChIP-seq in K562**
ENCSR000BRQ	CEBPB
ENCSR000DWE	CTCF
ENCSR000BLI	E2F6
ENCSR000BNE	EGR1
ENCSR000BMD	ELF1
ENCSR000BKQ	ETS1
ENCSR000BMV	FOSL1
ENCSR000BLO	GABPA
ENCSR000BKM	GATA2
ENCSR000EFV	MAX
ENCSR000BNV	MEF2A
ENCSR000BMW	REST
ENCSR000BKO	SP1
ENCSR000BGW	SPI1
ENCSR000BLK	SRF
ENCSR000BRR	STAT5A
ENCSR000BKT	USF1
ENCSR000BKU	YY1
ENCSR000BKF	ZBTB33
	**TF ChIP-seq in HepG2**
ENCSR000BID	BHLHE40
ENCFF002CTU	BRCA1
ENCFF002CTV	CEBPB
ENCSR000DUG	CTCF
ENCSR000BMZ	ELF1
ENCFF002CUA	ESRRA
ENCSR000BHP	FOSL2
ENCSR000BMO	FOXA1
ENCSR000BNI	FOXA2
ENCSR000BJK	GABPA
ENCSR000BLF	HNF4A
ENCSR000BNJ	HNF4G
ENCFF002CUD	HSF1
ENCSR000BGK	JUND
ENCFF002CUG	MAFF

ENCFF002CUI	MAFK
ENCFF002CUJ	MAX
ENCFF002CUY	NR2C2
ENCFF002CUM	NRF1
ENCSR000BOT	REST
ENCFF002CUT	RFX5
ENCSR00BHU	RXRA
ENCSR000BJX	SP1
ENCSR000BOU	SP2
ENCFF002CUV	SREBF1
ENCFF001VLB	SREBF2
ENCSR000BLV	SRF
ENCFF002CUW	TBP
ENCSR200BJG	TCF12
ENCFF002CUX	TCF7L2
ENCSR000BGM	USF1
ENCFF002CUZ	USF2
ENCSR000BHR	ZBTB33
	**TF ChIP-seq in H1-hESC**
ENCFF002CQQ	BRCA1
ENCFF002CQR	CEBPB
ENCFF002CIU	CTCF
ENCFF002CIV	EGR1
ENCFF002CIW	FOSL1
ENCFF002CIX	GABPA
ENCFF002CQU	JUN
ENCFF002CQY	JUND
ENCFF002CQZ	MAFK
ENCFF002CRA	MAX
ENCFF002CRC	NRF1
ENCFF002CJB	REST
ENCFF002CRE	RFX5
ENCFF002CJH	RXRA
ENCFF002CJK	SP1
ENCFF002CJL	SP2
ENCFF002CJN	SRF
ENCFF002CRH	TBP
ENCFF002CJQ	TCF12
ENCFF002CJS	USF1
ENCFF002CRI	USF2
ENCFF002CJT	YY1
	**TF ChIP-seq in GM12878**
ENCFF002CGQ	BATF

ENCFF002CGU	CEBPB
ENCFF002CGV	EBF1
ENCFF002CGW	EGR1
ENCFF002CGX	ELF1
ENCFF002CGY	ETS1
ENCFF002CGZ	FOXM1
ENCFF002CHA	GABPA
ENCFF939TZS	JUNB
ENCFF002CHC	MEF2A
ENCFF002CHH	REST
ENCFF002CHT	RXRA
ENCFF002CHV	SP1
ENCFF002CHQ	SPI1
ENCFF002CHW	SRF
ENCFF002CHX	STAT5A
ENCFF002CHZ	TCF12
ENCFF002CIA	TCF3
ENCFF144PGS	TCF7
ENCFF002CIB	USF1
ENCFF002CIC	YY1
ENCFF694OTE	ZBED1
ENCFF002CID	ZBTB33
ENCFF002CIE	ZEB1

Supplementary Table 4: ENCODE accession numbers of TF ChIP-seq data.

3 Position specific energy matrices (PSEMs)

Our current collection of PSEMs is comprised of matrices from JASPAR [9], HOCOMOCO [10], and the Kellis ENCODE Motif database [11].

In detail, the current collection contains from the JASPAR 2018 Core database:

- 579 PSEMs for vertebrates,
- 176 PSEMs for fungi,
- 26 PSEMs for nematodes,
- 489 PSEMs for plants,
- 1 PSEM for urochordates,
- 133 PSEMs for insects.

Additionally, we provide species-specific collections of JASPAR matrices:

- 3 PSEMs for Antirrhinum majus,
- 5 PSEMs for Arabidopsis lyrata,
- 440 PSEMs for Arabidopsis thaliana,
- 22 PSEMs for Caenorhabditis elegans,
- 132 PSEMs for Drosophila melanogaster,
- 1 PSEM for Fragaria x ananassa,
- 7 PSEMs for Gallus gallus,
- 6 PSEMs for Glycine max,
- 1 PSEM for Halocynthia roretzi,
- 459 PSEMs for Homo sapiens,
- 1 PSEM for Hordeum vulgare,
- 1 PSEM for Medicago truncatula,
- 1 PSEM for Meleagris gallopavo,
- 157 PSEMs for Mus musculus,
- 1 PSEM for Neurospora crassa,
- 1 PSEM for Nicotiana,
- 4 PSEMs for Orcytolagus,
- 7 PSEMs for Oryza sativa,
- 1 PSEM for Petunia x hybrida,
- 1 PSEM for Phaeodactylum tricornutum,
- 9 PSEMs for Physcomitrella patens,
- 3 PSEMs for Pisum sativum,
- 1 PSEM for Populus trichocarpa,
- 2 PSEMs for Rattus norvegicus,
- 2 PSEMs for Rattus rattus,
- 176 PSEMs for Saccaromyces cerevisiae,
- 2 PSEMs for Solanum lycopersicum,
- 1 PSEM for Triticum aestivum,
- 4 PSEMs for Xenopus laevis,
- 8 PSEMs for Zea mays.

From HOCOMOCO we provide 402 motifs for Homo sapiens and 358 for mus musculus. The Kellis set contains 58 TF motifs.

Besides, we provide non-redundant collections for homo sapiens and mus musculus considering motifs from all three sources:

- 561 PSEMs for homo sapiens,
- 380 PSEMs for mus musculus.

Furthermore, we used a motif clustering approach [12] to merge similar motifs of the aforementioned aggregated files. This led to:

- 483 PSEMs for homo sapiens,
- 306 PSEMs for mus musculus.

We generated PSEMs from position count matrices (PCMs) using the following conversion: A PCM *M* is converted to a PSEM E according to:

$E_{i,j} = \frac{1}{\lambda} log\left(\frac{M_{max,j}}{M_{i,j}} b_{i,j}\right)$, with $M_{max,j} = \max_{i \in \{A,C,G,T\}} M_{i,j}$.

The parameterλ is used for scaling the mismatch energies and $b_{i,j}$ denotes the background frequency of the nucleotide *i* with respect to the most frequent nucleotide at position j. By definition, if *j=max*, than $E_{i,j}$ = 0, as there should be no mismatch energy for the best possible sequence match. Note that, during conversion, a pseudo count *pc = 1* is added to each $M_{i,j}$. The conversion is done by a C++ tool provided by the authors of TRAP. This is also included in the TEPIC repository. As suggested in [13], we use the following parameters for the conversion:

λ =0.7, m=0.584, and n=-5.66.

The parameters slope *m* and intercept *n* are used to compute a matrix specific parameter R_0 that combines the concentration of the corresponding TF and the equilibrium constant of the binding reaction with its optimal binding site as defined in [13]. The authors of TRAP found a linear approximation for R_0 with:

$$\ln(R_0) = m \cdot |M| + n,$$

where *|M|* denotes the length of the PCM.

Further, we exploit species-specific GC-content values:

- homo sapiens = 0.41,
- mus musculus = 0.42,
- rattus norvegicus = 0.42,
- drosophila melanogaster = 0.43,
- caenorhabditis elegans = 0.36.

In all other cases, a default GC-content of 0.42 is used.

4 Score computation in TEPIC

TEPIC computes TF affinities using TRAP [13]. Extensive details on the mathematical background of TRAP can be found in Roider et al. [13]. Here, we only provide a brief summary of Section 2.3 of the aforementioned paper, which is extracted from the Supplement of [14]. In TRAP, one assumes that the fraction of TFs bound to a certain genomic location *S* is at an equilibrium such that the fraction of bound sites *p(S)* can be denoted as

$$p(S) = \frac{K(S)*[TF]}{1+K(S)*[TF]}.$$

Here, K denotes a site-specific equilibrium constant, which depends on the site with highest affinity (S_0), a TF specific mismatch energy *E(S)* and the Boltzmann constant k_B:

$$K(S) = K(S_0)e^{-\beta E(S)}.$$

Thus, we can denote p(S) as:

$$p(S) = \frac{K(S_0) * [TF] * e^{-\beta E(S)}}{1 + K(S_0) * [TF] * e^{-\beta E(S)}} = \frac{R_0 * e^{-\beta E(S)}}{1 + R_0 * e^{-\beta E(S)}}.$$

The mismatch energy E(S) is computed using a TF motif matrix according to:

$$\beta E(S) = \frac{1}{\lambda} \sum_{i=1}^{W} \sum_{\alpha=A,C,G,T} S_i^\alpha \log(\frac{m_{i,max}}{m_{i,\alpha}} b_{i,\alpha}).$$

Here, S_i^α, is an indicator function evaluating to 1 if the considered sequence S has letter α at position i. The most frequent element in the motif matrix is denoted by $m_{i,max}$. The parameter λ is a parameter used to scale the mismatch energy.

Thus, there are only two sequence and TF independent parameters R_0 and λ. For details on how these parameters are determined, please consult Sections 2.3 and 3.1 of Roider et al. [13].

Overall, TRAP computes the expected number *N* of TFs bound to sequence *S* with length *L* by summing up the binding score for each individual binding site in *S*:

$$N = p(S) = \sum_{l=1}^{L-W} p_l = \sum_{l=1}^{L-W} \frac{R_0 * e^{-\beta E_l(\lambda)}}{1+R_0 * e^{-\beta E_l(\lambda)}}.$$

Here, W denotes the length of the motif for the TF of interest.

Using our collections of PSEMs, TRAP computes TF binding affinities as described above in all user provided regions that could be found in the reference genomes of the respective species. To reduce run-time, the annotation can be further limited to only those genes overlapping with a window of user-defined size w centered at the most 5' TSS of each annotated gene in the considered organism.

Then, TF gene scores are computed by incorporating all candidate binding sites within the window centered on the 5' TSS of genes in the final score $a_{g,i}^w$. The contribution of the individual sites is weighted by their distance to the selected TSS with an exponential decay function [15].

Formally, the TF gene score $a_{g,i}^w$ for gene *g* and TF *i* is computed as

$$a_{g,i}^{w} = \sum_{p \in P_{g,w}} a_{p,i} e^{-\frac{d_{p,g}}{d_0}},$$

where $a_{p,i}$ is the affinity of TF *i* in peak *p*, the set $P_{g,w}$ contains all open-chromatin peaks in a window of size *w* around gene *g*, $d_{p,g}$is the distance from the center of peak p to the TSS of gene *g*, and d_0 is a constant fixed at 5kb. Additionally, affinities can be normalized by peak (and motif)-length during the computation of TF gene scores:

$$a_{g,i}^{w} = \sum_{p \in P_{g,w}} \frac{a_{p,i}}{|p|-|m_i|} e^{-\frac{d_{p,g}}{d_0}},$$

where */p/* is the length of peak *p*, $|m_i|$ is the length of the motif of TF *i*, with a pseudo-count of 1. If the signal within a peak should be directly considered in the TF gene score, we compute:

$$a_{g,i}^{w} = \sum_{p \in P_{g,w}} \frac{a_{p,i}}{|p|-|m_i|} s_p e^{-\frac{d_{p,g}}{d_0}},$$

where $s_p$is the per base signal in peak *p.* This computation can be performed with and without length normalization of the affinities. In addition to the TF gene scores, TEPIC can compute features for peak length (pl_g), peak count (pc_g), and peak signal (ps_s) following the same scoring formulation as for TF affinities:

$$pl_g = \sum_{p \in P_{g,w}} |p| e^{-\frac{d_{p,g}}{d_0}},$$

$$pc_g = \sum_{p \in P_{g,w}} e^{-\frac{d_{p,g}}{d_0}},$$

$$ps_g = \sum_{p \in P_{g,w}} s_p e^{-\frac{d_{p,g}}{d_0}},$$

where */p/* is the length of *p*. These features can be used for example to assess the influence of chromatin accessibility on gene expression without considering TF binding predictions. Furthermore, TEPIC can compute a TF specific affinity cut-off derived from either user-defined, or randomly generated sequences (*r*), to distinguish likely bound sites from unbound sites. Specifically, we compute TF affinities $a_{i,r}$ in those regions r to determine a TF specific cut-off, i.e. for TF *i* for the original affinities $a_{i,o}$using the frequency distribution of TF affinities $a_{i,r}$. As described in [16], TF affinities are normalized according to the length of their respective region:

$$a'_{i,r} = \frac{a_{i,r}}{|r|}, a'_{i,o} = \frac{a_{i,o}}{|o|}.$$

Using a p-value cut-off of 0.05 we determine a TF specific affinity threshold t_i from $a'_{i,r}$ and from this infer a binary TF to gene assignment $b_{i,o}$ according to

$$b_{i,o} = \begin{cases} 1, a'_{i,o} \geq t_i, \\ 0, a'_{i,o} < t_i. \end{cases}$$

These scores can be used to come-up with a binary TF gene assignment as described in [16].

5 Runtime evaluation of TEPIC

We benchmarked the runtime of TEPIC and its competitors using the Unix time utility (*/usr/bin/time*) on a compute server equipped with Intel Xeon CPU E7-8837 processors and 1TB of main memory. For TEPIC and JAMM we used 16 cores. Runtime was assessed for the original version of TEPIC, referred to as TEPIC1, for TEPIC2, as well as two conceptually different competitors FIMO-Prior [17] and PIQ [18]. We've computed TF affinities for the original PWM set comprising 458 TFs.

As shown in Figure 2, TEPIC2 improves considerably over the runtime of the original TEPIC implementation and is also outperforming both Fimo-Prior and PIQ considerably. The long-runtime of the latter two is due to two main reasons. Firstly, none of the methods comes with an easy-to-use build-in parallelization procedure and secondly, instead of reducing the annotation to several candidate sites, these tools screen the entire genome first to identify TF binding sites. In Supplementary Table 5, all runtimes are listed also including the runtime for peak calling with JAMM. Note that even with the time required for peak calling, TEPIC2 is still considerably faster than the competing methods.

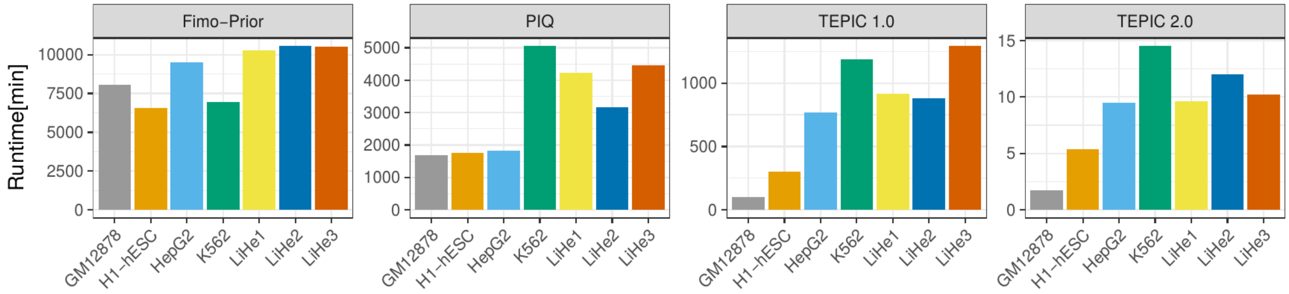

Supplementary Figure 2: Runtime comparison between Fimo-Prior, PIQ, TEPIC1 and TEPIC2. Note that the scale of the y-axis is different for each method.

**Sample\Runtime[min]**	PIQ	Fimo-Prior	TEPIC1	TEPIC2	TEPIC1+JAMM	TEPIC2+ JAMM
HepG2	1836	9480	765	10	960	205
GM12878	1685	8046	100	2	358	260
H1-hESC	1748	6576	300	5	546	251
K562	5049	6942	1185	15	1386	216
LiHe1	4223	10272	915	10	1092	187
LiHe2	3167	10542	880	12	1002	134
LiHe3	4452	10506	1290	10	1608	328

Supplementary Table 5: Overview on the runtime of TEPIC2 compared to TEPIC1 with and without Peak calling using JAMM.

We used default parameters for PIQ by adapting the included shell scripts to be used with our data. Aside from the file paths, we did not change the settings. In Fimo-Prior, we increased the *max-stored-scores* to 200 000, instead of the default value. Specifically, the following commands have been used for the time measurement (each exemplified for one sample):

TEPIC1:

bash TEPIC.sh -g hs37d5.fa -b JAMM/41/LiHe/01/peaks/filtered.peaks.narrowPeak -o Time_Asses_Hf01 –p pwm_vertebrates_jaspar_uniprobe_converted.txt -a gencode.v19.protein_coding_only.gtf -c 16

TEPIC2:

bash TEPIC.sh -g hs37d5.fa -b JAMM/41/LiHe/01/peaks/filtered.peaks.narrowPeak -o Time_Asses_Hf01 –p pwm_vertebrates_jaspar_uniprobe_converted.PSEM -a gencode.v19.protein_coding_only.gtf -f gencode.v19.protein_coding_only.gtf -c 16

PIQ:

Execute the shell script shown below, adapted from the provided script *PIQ_1_3/testers/runall.k562.s*h

```
#!/bin/bash
jobname="hg19k562"
bampath="/MMCI/MS/DEEP-liver/work/Data/K562/DNase/BAMs/ENCFF441RET.bam"
tmpdir="/MMCI/MS/DEEP-liver/nobackup/PIQ_Temp"
basedir="/MMCI/MS/DEEP-liver/work/Tools/PIQ_1_3"
baseoutdir="/MMCI/MS/DEEP-liver/work/Tools/PIQ_1_3/Predictions_Paper/K562"
mkdir ${baseoutdir}
pushd $basedir
jobid="$(date +"%y%m%d")"
idname="$jobid-$jobname"
popd
commonfile="$basedir/common.r"
outdir="$baseoutdir/$idname.calls/"
bamfile="$baseoutdir/rdata/$jobname.RData"
jaspardir="$basedir/pwms/pwm_vertebrates_jaspar_uniprobe_converted.txt"
pwmdir="$baseoutdir/$idname.pwms/"
mkdir ${baseoutdir}"/rdata"
pushd $basedir
./bam2rdata.r $commonfile $bamfile $bampath
popd
mkdir $pwmdir
IDs=$(seq 458)
for pwmid in $IDs
do
    Rscript pwmmatch.exact.r $commonfile $jaspardir $pwmid $pwmdir
done
cp $jaspardir $pwmdir
cp $commonfile $pwmdir
mkdir $outdir
for pwmid in $IDs
do
    echo Rscript pertf.r $commonfile $pwmdir $tmpdir $outdir $bamfile $pwmid
    Rscript pertf.r $commonfile $pwmdir $tmpdir $outdir $bamfile $pwmid
done
```

Fimo-Prior:

./create-priors sequences_50000.fa 41Hf01.wig --parse-genomic-coord --oc Priors/41Hf01_50000

and

./fimo --oc T41_01_50000_1 --psp Priors/41Hf01_50000/priors.wig --prior-dist Priors/41Hf01_50000/priors.dist pwm_vertebrates_jaspar_uniprobe_converted.meme sequences_50000.fa --max-stored-scores 200000

Peak-Calling with JAMM:

bash JAMM.sh -s 41_Hf01.bed -o Peaks/41_Hf01 -g hg19.genomseSize.txt -f 1 -p 16

When applicable, we reduced the annotation to a 50kb area around the TSS's of the genes to be annotated*.* This is not possible with the original TEPIC version and also not with PIQ.

6 Assessment of TFBS predictions using ChIP-seq data

We obtained TF ChIP-seq data from ENCODE for HepG2, K562, GM12878, and H1-hESC in narrow peak format. All downloaded files are listed in Sup. Tab. 4. We use the same validation strategy as in [8], which defines the gold-standard set to be composed of all motif predicted binding sites that overlap with a ChIP-seq peak of the respective TF and all other motif predicted sites as negatives. We used Fimo with JASPAR matrices to screen the genome for TF binding sites and considered all sites with a p-value < 0.05. Thereby, we generated a gold-standard set for 33 TFs in HepG2, 19 TFs in K562, 24 TFs in GM12878, and 22 TFs in H1-hESC.

Consequently, we define a true positive site (TP) as a site predicted with a score > the current threshold overlapping with the gold-standard, a false positive (FP) as a site predicted with a score > the current threshold not overlapping with the gold-standard, a true negative (TN) is a site of the negative set which is either not overlapping any predicted site or having a score ≤ the current threshold. A false negative (FN) is defined as a site of the gold-standard set not overlapping with our predictions.

Because this definition of an evaluation scheme leads to an unbalanced set of true and negative sites, i.e. there are many more negative than positive sites, we use area under the precision recall curve (AUPR) as a performance measure, since the more common ROC-curves would be biased towards the negative set, overestimating the performance of the models. Additionally, computing precision-recall (PR)-curves has the advantage that no threshold needs to be chosen for the TFBS prediction itself, as the entire scope of possible thresholds is sampled and precision and recall are computed accordingly.

We have limited ourselves to compare TEPIC against Fimo -Prior and PIQ for several reasons: One of the first methods that successfully combined epigenetics data with the sequence dependence of TFs was CENTIPEDE, by Pique-Regi et al, and was considered as a gold-standard for TFBS-prediction methods in the field. Therefore, many tools have been compared against it, including Fimo-Prior and PiQ. The first one, Fimo-Prior, is essentially a simplification of CENTIPEDE, designed to avoid the potential overfitting of CENTIPEDEs mixture model and including only one epigenetic signal, e.g. DNase1-seq [17]. Additional features, for example sequence-conservation, or additional histone marks are not considered. The authors of Fimo-Prior have shown that, although their method is less complex and easier to use then CENTIPEDE, it does perform almost on par with the CENTIPEDE model [17].

The second method, PIQ, is based on Bayesian inference to predict TFBS and was used to identify pioneering TFs, which open up the chromatin [18]. The authors of PIQ showed that their method performs favorably compared to Centipede as well as compared to DGF [31] another method considering DNase1-seq data. The observation of Sherwood et al., that PIQ outperforms CENTIPEDE is reflected in our evaluation as well, since PIQ is also outperforming Fimo-Prior.

Due, to those findings, we decided not to include either CENTIPEDE nor DGF in our evaluation, as both Fimo-Prior and PIQ have been shown to perform on par or better than CENTIPEDE and DGF before, and are more prevalently used in practice.

Furthermore, we excluded both Millipede and BinDNase, which was shown before to be at least as reliable as or better than Millipede [28], from consideration because these are supervised methods that require TF ChIP-seq data to be used for model training. Since this drastically reduces the applicability of these methods in practice, we did not consider them.

However, we did check for the potential performance of these methods, compared to our tool and compared to Fimo-Prior and PIQ. We found that both Millipede and BinDNase outperform CENTIPEDE and that BinDNase slightly outperforms PIQ on a few TFs [28]. However, we do note that this comparison is (1) between an unsupervised and a supervised method, and therefore not completely fair. Also (2), the comparison is presented only on a negative set, which might be overestimating model performance due to an unbalanced distribution of negative and positive sites. Furthermore (3), we were unable to obtain an implementation of the BinDNase approach, as the link provided in the paper is not working.

Recently published DEEP learning approaches, such as DEEP-Bind [45] have not been considered either as they require large amounts of data to be used, special hardware which might not be present in each lab, and are hard to interpret. The easy availability of PWMs, and their straight forward interpretation still renders them to be the most common way of describing sequence preferences of TFs, explaining why they are frequently used in practice.

Purely sequence based approaches, as listed in Sup. Table 1 have not been considered as they were shown to have a high-false positive rate with genome wide TFBS predictions [17].

Here, we used *bedtools* to intersect the predictions from TEPIC, Fimo-Prior, and PIQ with our gold-standard set. These resulting files are reformatted with custom python scripts to be usable as input for the PRROC R-package [19]. The PRROC package uses continuous interpolation to compute PR-curves from both soft and hard-labeled data (as present in our setting). Based on the PR-curves, the package computes the AUPR values for each TF and each TFBS prediction method. Details on the required input format can be found in the PRROC documentation.

Internally, the PRROC package computes Precision (Pre) as

$$PR = \frac{TP}{TP+FP},$$

and Recall (Rec) as

$$Rec = \frac{TP}{TP+FN}.$$

Here, we compared TF affinities computed in TF footprints identified with HINT against TFBS predictions from Fimo-Prior [17] and PIQ[18] using JASPAR TF motifs for all methods. We used the same command arguments as shown above for the runtime experiments, except that, for TEPIC we used footprint calls as input and computed predictions with TEPIC and Fimo-Prior genome-wide:

TEPIC2:

bash TEPIC.sh -g hs37d5.fa -b HepG2_Footprints.bed -o HepG2_HINT –p pwm_vertebrates_jaspar_uniprobe_converted.PSEM -c 16

Fimo-Prior:

./create-priors hg19.fa HepG2.wig --parse-genomic-coord --oc Priors/HepG2

and

./fimo --oc CHepG2 --psp Priors/HepG2/priors.wig --prior-dist Priors/HepG2/priors.dist pwm_vertebrates_jaspar_uniprobe_converted.meme hg19.fa --max-stored-scores 200000

Figure 1b shows aggregated AUPR values across several TFs per method and cell-line, while Supplementary Figure 3 shows the results for individual TFs and cell-lines. Overall, TF affinities combined with footprints outperform the other two methods for most TFs, however for some factors, e.g. NRF1 or REST, Fimo-Prior performs best. We also observe that the prediction qualities differ between cell-types, which might be linked to the quality of the available chromatin accessibility data.

We note that these performance measurements could have also been achieved with TEPIC 1.0, because the definition of the affinity computation has not been altered by switching the implementation of TRAP from R to C++.

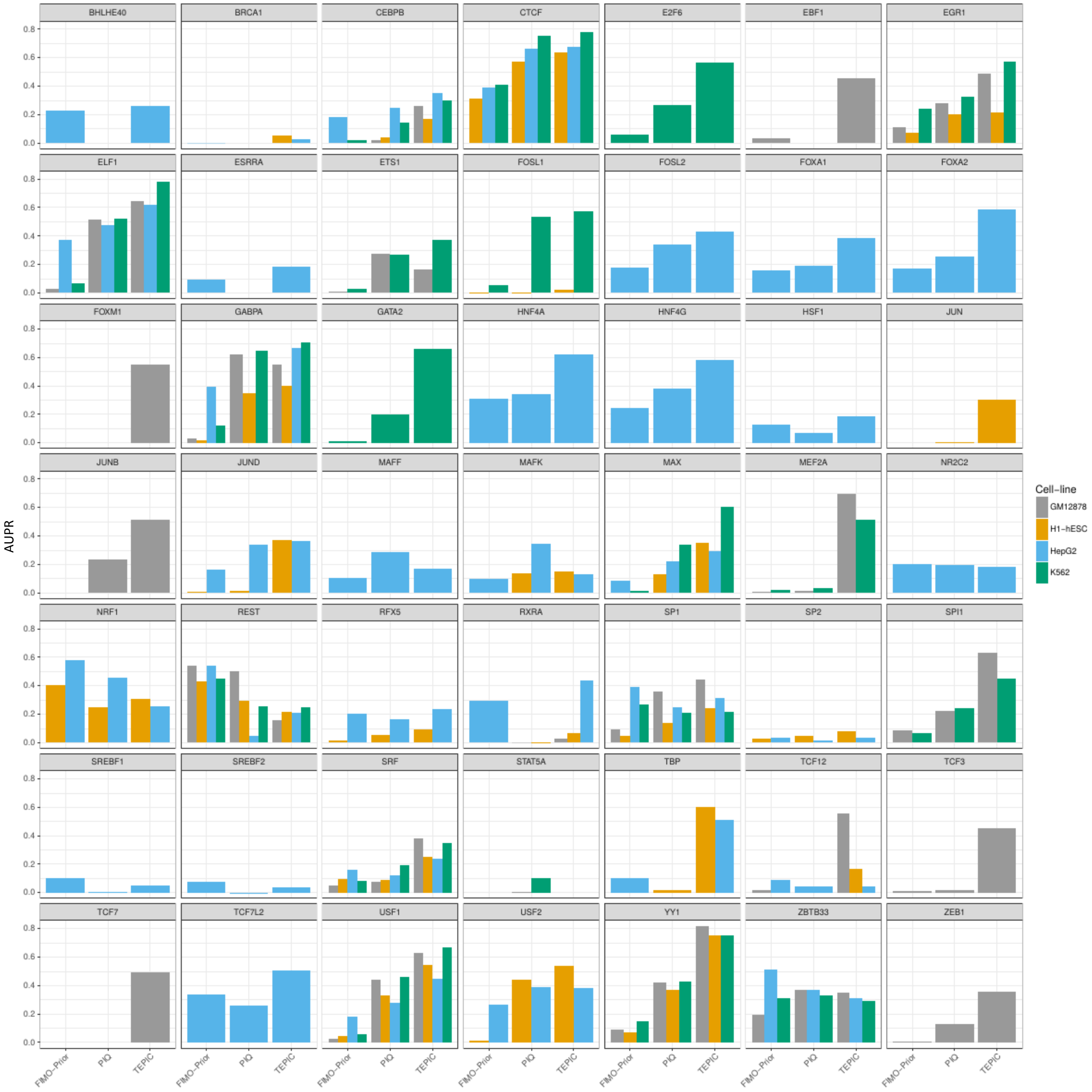

Supplementary Figure 3: TF and method specific AUPR values computed using PRROC [19].

7 Inferring essential regulators using linear regression (INVOKE)

7.1 Motivation

Epigenetics data contains a wealth of information on gene regulation. It was shown that especially data on open-chromatin is well suited to build predictive models of gene expression [20, 21, 22]. Interpreting these models allows the inference of regulators that may play a key role in gene expression regulation.

Here, we offer an integrated analysis of epigenetics data, e.g. open-chromatin data (DNase1-seq, ATAC-seq, NOMe-seq) and gene expression data to suggest key transcriptional regulators in the analyzed sample.

Note that, although incorporating epigenetic data greatly improved the performance of TF binding predictions, both computing TF binding predictions and linking TFs to genes are still unsolved problems and all predictions should be seen as suggestions and not as the absolute truth.

7.2 Description of the pipeline

The INVOKE analysis is split up into two main steps.

- Computing TF gene scores on the basis of epigenetic data using TEPIC (see Section 4).
- Learning a linear regression model to predict gene expression from TF gene scores computed in the previous step.

In order to learn about potentially important regulators, we build a linear, interpretable regression model, comparable to methods proposed in [20, 21, and 22].

Here, we use TF gene scores computed with TEPIC as features in a linear regression setup to predict gene expression. In such a per sample approach, we stick to the simplifying assumption that all genes are regulated similarly.

Features with a high regression coefficient can be seen as potential key regulators in the analyzed sample, as they seem to affect the expression of a large portion of the genes under consideration. However, the results of this method should be seen as suggestions for possible regulators and not as the absolute truth.

We offer three different regularization techniques:

Lasso:

$$\hat{\beta} = \underset{\beta}{\operatorname{argmin}}(\|y - X\beta\|^2 + \|\beta\|).$$

Ridge:

$$\hat{\beta} = \underset{\beta}{\operatorname{argmin}}(\|y - X\beta\|^2 + \|\beta\|^2).$$

Elastic net:

$$\hat{\beta} = \underset{\beta}{\operatorname{argmin}}(\|y - X\beta\|^2 + \alpha\|\beta\|^2 + (1-\alpha)\|\beta\|),$$

where β represents the regression coefficient vector, $\hat{\beta}$ represents the estimated coefficients, *X* is the feature matrix, *y* is the response vector, and the parameter α controls the distribution between Ridge and Lasso penalty in the elastic net.

Using Lasso regularization, models are sparse and can be learned very fast. However, Lasso cannot properly deal with correlated features, e.g. instead of distributing the coefficients among them, only one is selected. Also, Lasso solutions are not stable and therefore should be interpreted with caution. Nevertheless, Lasso regularization is good to get a first impression of model performance. The disadvantage of Ridge regression is that it cannot produce sparse models (many coefficients being exactly 0), which may hinder interpretability.

Elastic net regularization was designed to overcome the limitations of both regularization techniques mentioned above. It resolves the correlation between features by distributing the feature weights among them, and simultaneously leads to sparse and stable models [23]. However, training a model using elastic net penalty is slower than using either only Lasso or Ridge regularization.

In detail, the data matrix *X*, containing TF gene scores, and the response vector *y*, containing gene expression values, are log-transformed, with a pseudo-count of 1, centered and scaled. Regression coefficients are computed in an inner cross validation, the α parameter of elastic net regularization is optimized with a default step size of 0.1.

We offer two ways to use our learning pipeline:

1. Learn a model for feature interpretation without computing performance measures: In order to provide a time efficient way of obtaining an interpretable model and to prevent a potential loss of information by considering only a portion of the full data set for model training, the regression coefficients are determined on the entire data set.
2. Learn a model for feature interpretation and compute model performance: Nested cross-validation is used to learn the models and to assess their performance. Per default, 20% of the data are used as test data and 80% are used as training data. Model performance is assessed in an outer cross validation. We report the mean Pearson correlation, the mean Spearman correlation, and the mean squared error over the outer folds as measures of model performance. Additionally, a model is learned on the entire data set as described in (1) for interpretation of the coefficients.

All parameters mentioned in this section can be changed by the user. The training process is sketched in Sup. Fig. 4.

In addition to the input required for the computation of TF gene scores in TEPIC, a file containing gene expression data must be provided to run INVOKE. This file should be structured such that column 1 contains the gene identifiers and column 2 holds expression values.

An INVOKE analysis always provides the user with the following files:

- a list of regression coefficients computed on the entire data set,
- a bar plot showing the regression coefficients with an absolute value > 0.025.

The larger a regression coefficient, the stronger is the inferred effect of the corresponding TF on gene expression. Positive coefficients suggest an activating influence of TFs, negative coefficients suggest an inhibiting effect.

If model performance was assessed, the following is available as well:

- a summary on model performance containing the aforementioned measures (Pearson correlation, Spearman correlation, mean squared error),
- a list of regression coefficients determined in the outer cross validation,
- a heatmap visualizing the regression coefficients determined in the outer cross validation for at most the top 10 positive and negative features, sorted according to their median.
- an image showing a box plot for Pearson and Spearman correlation respectively.
- scatter plots showing the predicted vs the measured gene expression for each outer cross validation fold.

The heatmap can be easily used to judge model performance, as it shows the regression coefficients of all outer-cross validation runs. The box plots provide further insights into model performance and stability across the outer folds of the cross validation.

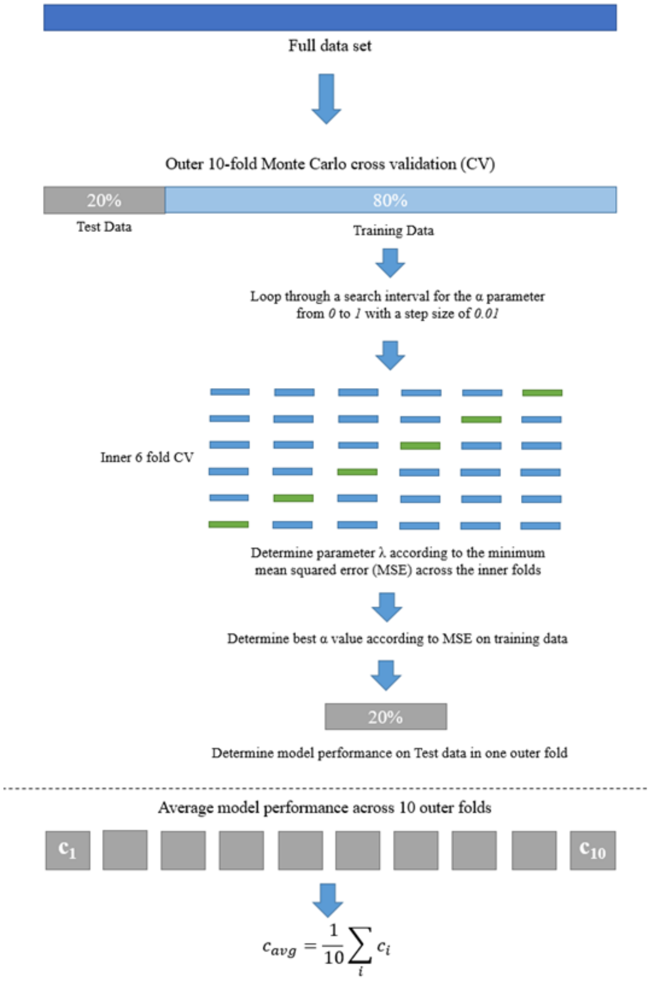

Supplementary Figure 4: A scheme of the machine learning paradigm used for INVOKE

7.3 Example

An example for INVOKE is included in the repository of TEPIC (https://github.com/SchulzLab/TEPIC). Here, we learn a predictive model for a macrophage sample from Blueprint (S001S7). To keep the runtime short, we consider only DHS sites from chromosome 1.

Running the example generates a bar-plot (Sup. Fig. 5) as well as a heatmap (Sup. Fig. 6) showcasing regression coefficients, scatter plots (Sup. Fig. 7) comparing measured against predicted gene expression per outer cross-fold as well as a boxplot showing model performance (Sup. Fig. 8). Note that the bar-plot reflects the model coefficients learned on the entire data-set, while the heatmap shows the coefficients determined during model training in the cross-validation procedure. Text files holding information on model performance and on the regression coefficients are provided as well.

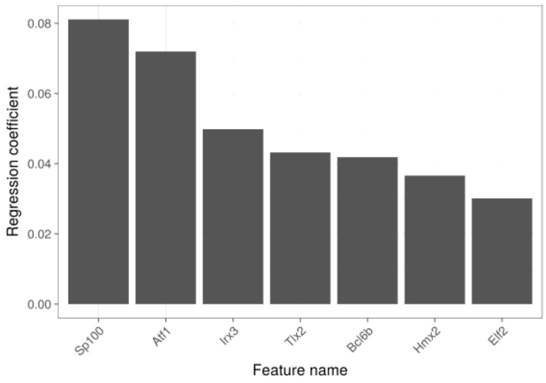

Supplementary Figure 5: Regression weights computed on the entire data sets. Only coefficients with an absolute weight > 0.025 are shown.

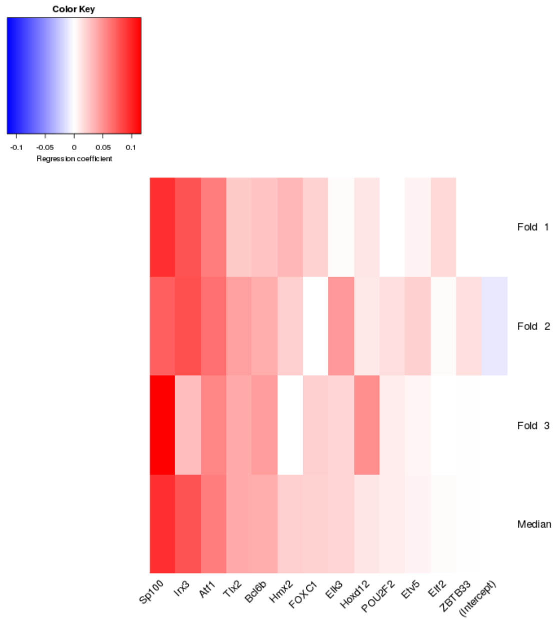

Supplementary Figure 6: Regression weights computed during the cross-validation procedure. At most the top 10 positive and negative coefficients are shown per fold, including their median value.

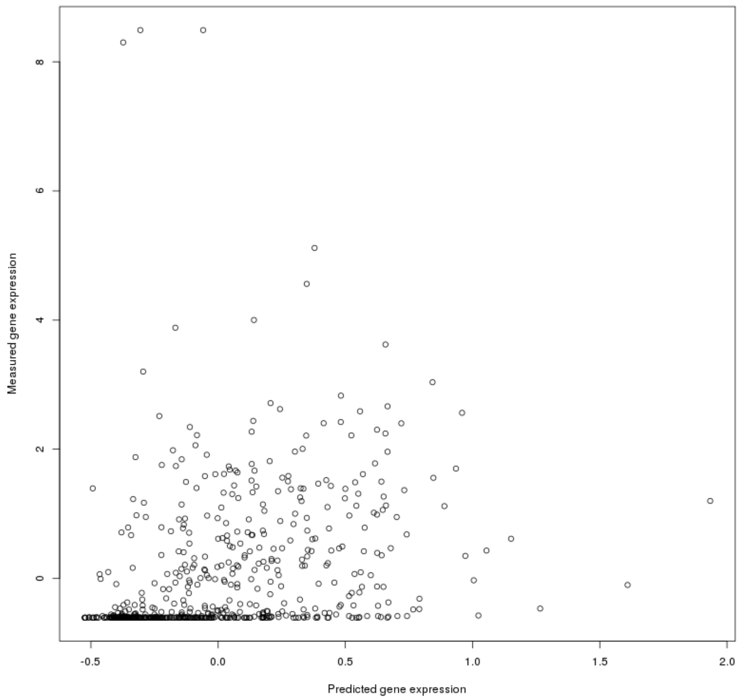

Supplementary Figure 7: Scatter plots contrasting predicted (x) versus measured (y) gene expression are generated for each outer-fold.

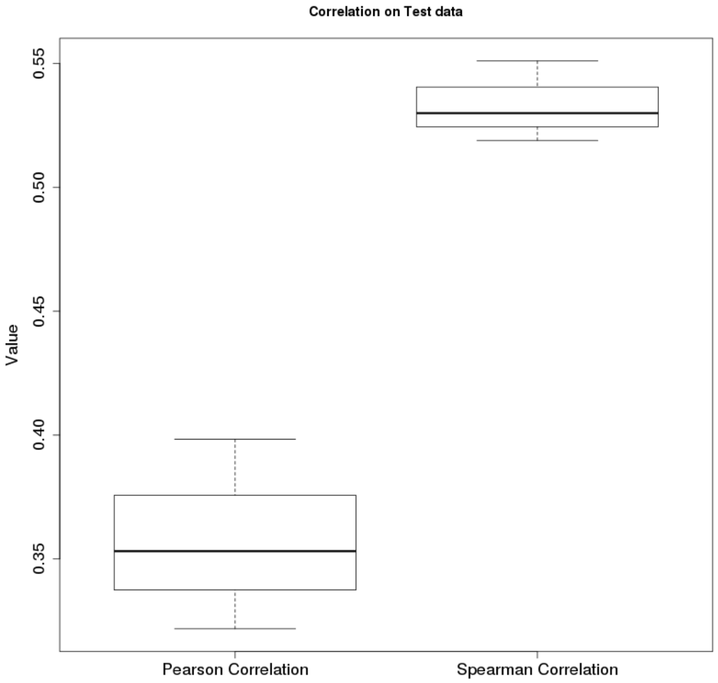

Supplementary Figure 8: Boxplots showing model performance computed in terms of Pearson and Spearman correlation across the outer cross-validation folds.

8 Determining regulator of differential gene expression (DYNAMITE)

8.1 Motivation

In addition to the INVOKE analysis, which highlights regulators associated to stable gene expression values measured within one sample, we propose a method to infer the most likely transcriptional regulators for a set of differentially expressed genes.

We use TF scores, computed using TEPIC, and logistic regression to identify TFs that have explanatory power to distinguish between up- and down-regulated genes, e.g. between to samples or between healthy and diseased tissue.

8.2 Description of the pipeline

To run DYNAMITE, a user must provide candidate regions of TF binding for two groups of samples, A and B, e.g. control and disease. These can be derived, for example, by open chromatin experiments such as DNase-seq. It is essential that the candidate regions reflect the characteristics of chromatin organization in the analyzed tissues. In addition, a list of differential expressed genes between two groups as well as log2 fold changes of the expression are needed.

Our method consists of two parts: (1) TF gene score computation, and (2) identification of key TFs.

1. Computing TF gene scores:
   Using TEPIC, we compute TF gene scores g_{ij} for all differentially expressed genes *i* and distinct TFs *j* considering the provided candidate regions for all replicates a of group A and for all replicates b of group B. As a result, gene-TF matrices M_k for all replicates of both groups are obtained. To account for biological variation among the replicates, we compute two matrices M_A, M_B holding the mean TF gene scores among all replicates of a group, where

   $$M_{A_{ij}} = \frac{\sum_{a \in A} a_{ij}}{|A|}, M_{B_{ij}} = \frac{\sum_{b \in B} b_{ij}}{|B|}.$$

   Using matrices M_A and M_B we compute a matrix R_{AB} that holds the ratios of TF gene scores for all genes and all TFs:

   $$R_{AB_{ij}} = \frac{M_{A_{ij}}}{M_{B_{ij}}}.$$

   Thus, R_{AB} represents the changes in TF binding between groups A and B on a gene level. The feature computation is sketched in Sup. Fig. 9.

2. Identification of potential key TFs:

   To identify those TFs that can explain the differential expression state of as many genes as possible, we build a logistic regression classifier. We use matrix R_{AB} computed in Step 1 as the feature matrix X, and a binary vector of gene expression changes as response y. An example is shown in Sup. Fig. 10.

   We perform logistic regression with elastic net regularization [23]. As above, we tune the parameter α that distributes the weight between lasso and ridge penalty in a grid search with user-defined step-size between 0 and 1. Model parameters are learned in an inner cross

validation, while the accuracy of our classifier can be assessed through an outer cross validation. This is the same machine learning paradigm that is described for the INVOKE analysis (Sup. Fig. 4). We use the entire dataset for model training and to interpret the regression coefficients. TFs that correspond to features with a non-zero regression coefficient can be seen as being essential to explain the observed expression differences and should be further investigated.

Model performance is reported in a text file and visually in a bar plot using mean test and training accuracy as well as the F1 measure. A heatmap shows the regression coefficients in the outer cross validation folds. Additionally, we report confusion matrices for the outer cross validation folds. We generate a bar-plot with the regression coefficients of all TFs selected in the final model. A positive coefficient is used by the model to predict genes as upregulated, a negative coefficient is related to genes that are predicted as downregulated. The interpretation of the model can be simplified if the user makes sure that both TF ratios and gene expression fold changes are computed in the same order.

We provide an additional script to generate further plots per feature that can help to understand the model. As shown in Sup. Fig. 11 density and scatter plots are generated to help elucidating why a particular feature was selected by the model.

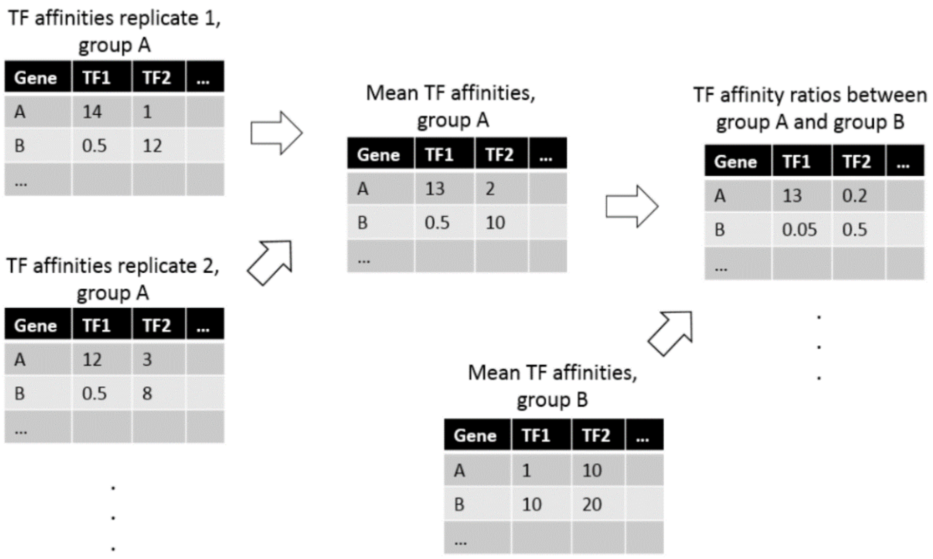

Supplementary Figure 9: Computation of differential TF features between two groups.

Gene	Expression Changes	TF1	TF2	...
A	Up	1.2	3.9	
B	Down	4.2	0.7	
C	Down	0.8	1.7	
D	Up	0.4	1.6	
E	Up	1.0	1.2	
...				

Supplementary Figure 10: Example for a matrix used as input to the logistic regression. The column Expression Changes is used as response, while the affinity ratios TF_x are used as features.

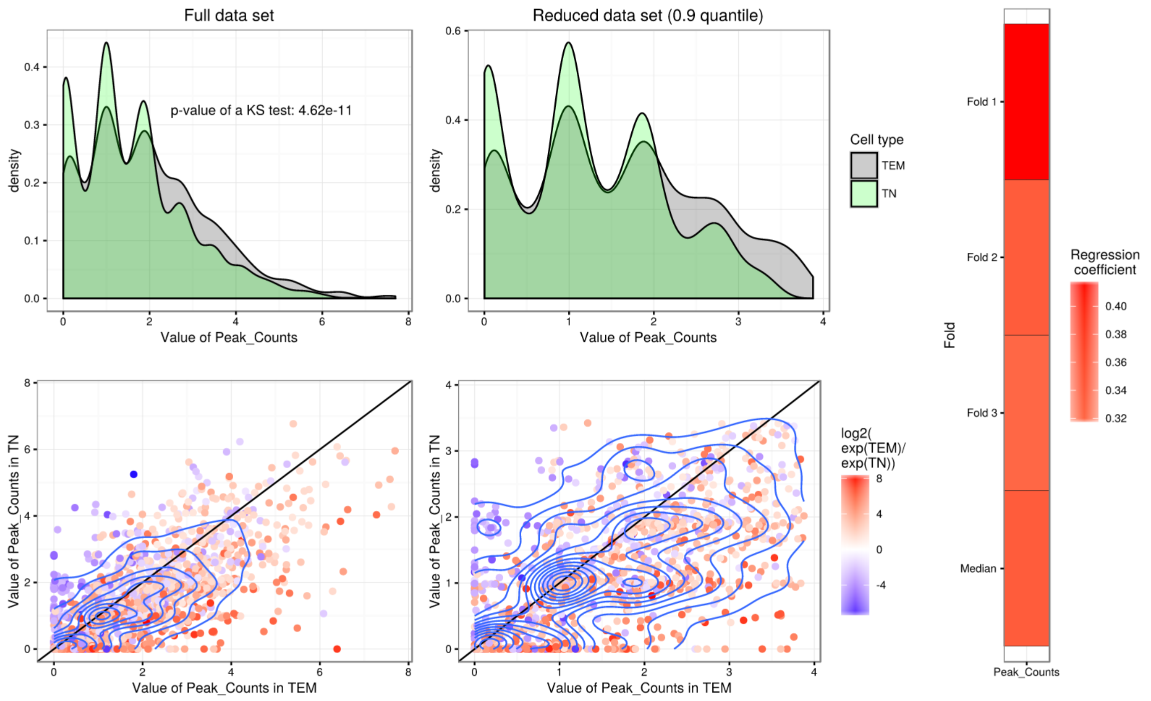

Supplementary Figure 11: Example for an automatically created feature analysis Figure generated on the example data provided in the repository. The density plots show the distribution of TF affinities, the scatter plot relates the TF affinities to the observed expression changes. The miniature heatmap shows the regression coefficients determined during the outer cross validation.

8.3 Example

As for INVOKE, we provide an example for DYNAMITE in the TEPIC repository. Here, we investigate gene expression differences between two T-cell samples generated in scope of the DEEP project (Hf03_BlEM and Hf03_BlTN) using open chromatin regions identified with NOMe-seq.

DYNAMITE produces bar plots showing model performance (Sup. Fig. 12), bar plots showing regression coefficients learned on the entire data set (Sup. Fig. 13) or during the cross-validation procedure (Sup. Fig. 14). Using an additional script and the model results, an overview Figure as depicted in Sup. Fig. 11 can be computed for any feature the user is interested in. This can provide insights into how the features should be interpreted.

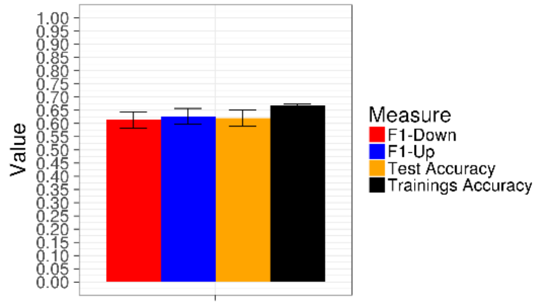

Supplementary Figure 12: Bar plots showing model performance in terms of F1-measure computed for Up-regulated genes, for down-regulated genes as well as accuracy computed for training and test data.

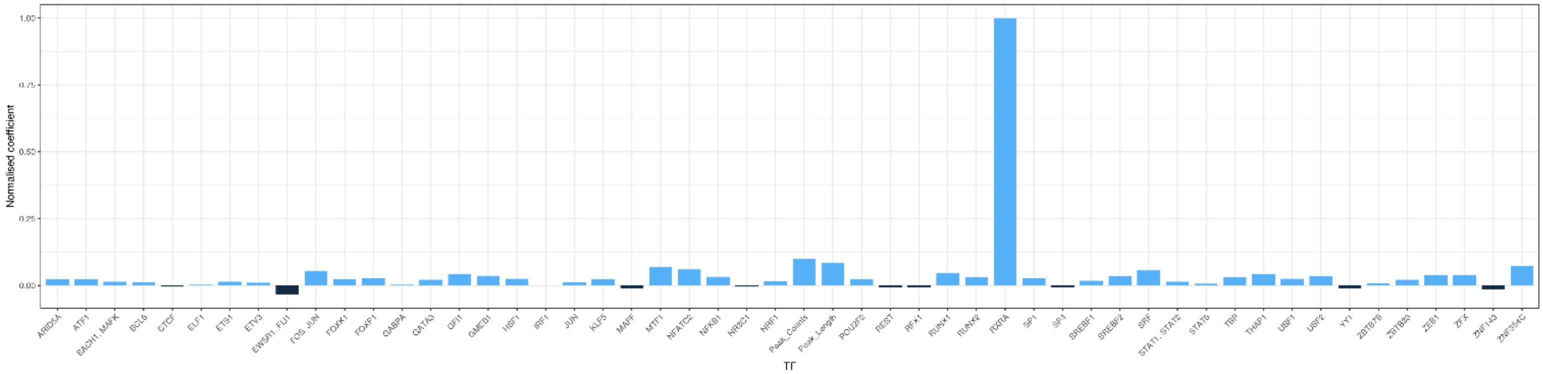

Supplementary Figure 13: Bar plot showing the value of regression coefficients computed on the entire data set.

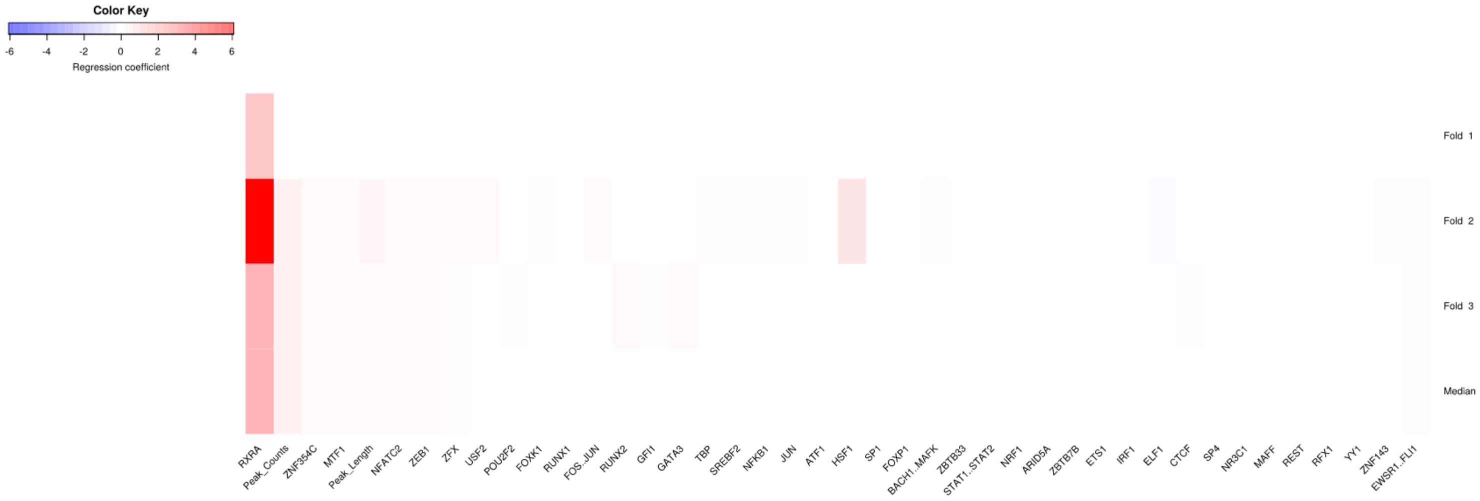

Supplementary Figure 14: Heatmap visualizing the regression weights of coefficients for the individual outer-cross folds.

9 Identification of important regulators from time series epigenomics and expression data (EPIC-DREM)

9.1 Motivation

EPIC-DREM is a combination of TEPIC and the *Dynamic Regulatory Events Miner (DREM)* [24]. Instead of using static ChIP-seq data, which is provided in DREM 2.0, we suggest to use time-point specific TF binding predictions based on time-dependent epigenomic profiles. Thereby, DREM can infer regulators that can be linked to expression changes at distinct points in time. We have shown that using the predicted, dynamic TF binding events is superior to the static data included in DREM [16].

9.2 Description

In order generate a sparse input matrix for DREM, we devised a strategy to threshold TF affinities based on a set of background sequences. These can be either chosen automatically or be provided by the user.

In Sup. Fig. 15, we illustrate how TF affinities can be discretized and illustrate their usage in DREM. Note that DREM is not included in the TEPIC repository. It is available online at http://www.sb.cs.cmu.edu/drem/.

In some applications it is required to make a binary decision whether a factor is binding or not to a distinct sequence. To infer this information from TF affinities, TEPIC allows the computation of a TF specific affinity threshold by calculating TF affinities on a randomly selected set of genomic regions. When selected by TEPIC, these regions show similar characteristics compared to the provided regions (GC content and length). Alternatively, a set of background regions can be provided by the user. By applying a user-defined p-value on the distribution of affinities computed on the random regions, a threshold is chosen. Per TF, all affinities that are smaller than the selected threshold, are set to zero, thus a sparse matrix with TF gene interactions can be generated.

In addition to the standard input required for TEPIC, a reference genome in 2bit format is needed if TEPIC should determine the background sequences automatically. Alternatively, the user needs to provide a bed file containing background regions.

In addition to the standard-output, this will generate:

1. TF affinities for all selected PSEMs in the regions provided by the user that passed the filtering step, where all affinities below the specific thresholds are set to 0.
2. (Length normalized) TF gene scores for all selected PSEMs calculated as described above (optionally including peak features) using the thresholded affinities.
3. A sparse representation of TF gene interactions.

Either (2) or (3) can be combined with RNA-seq data and used as input for DREM.

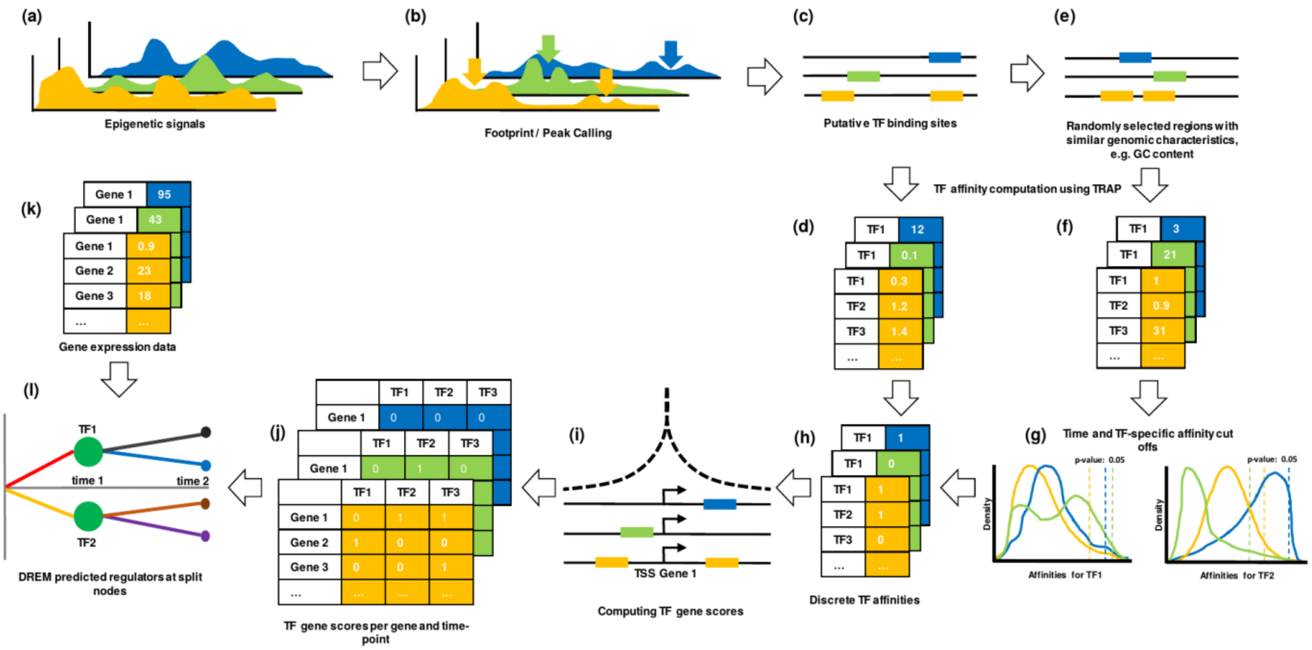

Supplementary Figure 15: Overview on the EPIC-DREM approach: Note that in this Figure, different time points are indicated by different colors. First, epigenetic data, e.g. DNase1 experiments, are conducted for different points (a). Next, putative TF binding sites are identified by peak and/or footprint calling (b, c) and annotated with TF affinities (d). From the putative binding sites, a random set of genomic regions is chosen (e) and annotated with TF affinities as well (f). By applying a p-value cut-off on the distribution of TF affinities calculated on the random regions (g), a suitable, TF specific affinity threshold is chosen to discretize the original TF affinities (h). Using the default TEPIC TF gene score formulation (i), a TF gene interaction matrix (j) is computed. Together with gene expression data (k), the sparse matrix (j) can be used as input for DREM (l) to identify potential key regulators of expression changes in time series data.

10 References

[0] Schmidt et al., Combining transcription factor affinities with open-chromatin data for accurate gene expression prediction, NAR, 2017.

[1] German epigenomics project (DEEP), http://www.deutsches-epigenom-programm.de/

[2] The ENCODE Project Consortium, An integrated encyclopedia of DNA elements in the human genome, Nature, 2012.

[3] Trapnell et al., TopHat: discovering splice junctions with RNA-seq, Bioinformatics, 2009.

[4] Langmead et al., Ultrafast and memory-efficient alignment of short DNA sequences to the human genome, Genome Biology, 2009.

[5] Trapnell et al., Differential gene and transcript expression analysis of RNA-seq experiments with TopHat and Cufflinks, Nature Protocols, 2012.

[6] Li et al., Fast and accurate short read alignment with Burrows Wheeler transform, Bioinformatics, 2009.

[7] Li et al., The Sequence alignment/map (SAM) format and SAMtools, Bioinformatics, 2009.

[8] Gusmao et al., Detection of active transcription factor binding sites with the combination of DNase hypersensitivity and histone modifications, Bioinformatics, 2014.

[9] Khan, A. et al. JASPAR 2018: update of the open-access database of transcription factor binding profiles and its web framework. Nucleic Acids Res. 2018.

[10] Kulakovskiy et al., HOCOMOCO: towards a complete collection of transcription factor binding models for human and mouse via large-scale ChIP-Seq analysis, Nucleic Acids Research, 2018.

[11] Kheradpour et al., Systematic discovery and characterization of regulatory motifs in ENCODE TF binding experiments, Nucleic Acids Res. 2013.

[12] Pape et al., Natural similarity measures between position frequency matrices with an application to clustering, Bioinformatics, 2008.

[13] Roider et al., Predicting transcription factor affinities to DNA from a biophysical model, Bioinformatics, 2007.

[14] Schmidt et al., On the problem of confounders in modelling gene expression, In Review.

[15] Ouyang et al., ChIP-Seq of transcription factors predicts absolute and differential gene expression in embryonic stem cells, Proc. Natl. Acad Sci. USA, 2009.

[16] Gerard et al., Temporal epigenomic profiling identifies AHR and GLIS1 as super-enhancer-controlled regulators of mesenchymal multipotency, Bioarxiv, 2017.

[17] Cuellar-Partida et al., Epigenetic priors for identifying active transcription factor binding sites, Bioinformatics, 2011.

[18] Sherwood et al., Discovery of directional and nondirectional pioneer transcription factors by modeling DNase profile magnitude and shape, Nature Biotechnology, 2014.

[19] Grau et al., PRROC: computing and visualizing precision-recall and receiver operating characteristic curves in R, Bioinformatics, 2015.

[20] Natarajan et al., Predicting cell-type-specific gene expression from regions of open chromatin, Genome Res. 2012.

[21] Budden et al., Predictive modelling of gene expression from transcriptional regulatory elements, Brief Bioinformatics, 2015.

[22] McLeay et al., Genome-wide in silico prediction of gene expression, Bioinformatics, 2012.

[23] Friedman et al., Regularization paths for generalized linear models via coordinate descent, Journal of statistical software, 2009.

[24] Schulz et al., Improved reconstruction of dynamic regulatory networks from time-series expression data, BMC Syst Biol., 2012.

[25] Pique-Regi et al., Accurate inference of transcription factor binding from DNA sequence and chromatin accessibility data, Genome Res., 2011.

[26] Grant et al., FIMO: scanning for occurrences of a given motif, Bioinformatics, 2011.

[27] Luo et al., Using DNase digestion data to accurately identify transcription factor binding sites, Pac. Symp. Biocomput., 2013.

[28] Kähärär et al., BinDNase: a discriminatory approach for transcription factor binding prediction using DNaseI hypersensitivity data, Bioinformatics, 2015.

[29] Ernst et al., ChromHMM: automating chromatin state discovery and characterization, Nat. Methods, 2012.

[30] Baek et al., Bivariate Genomic Footprinting Detects Changes in Transcription Factor Activity, Cell Reports, 2017.

[31] Neph et al., An expansive human regulatory lexicon encoded in transcription factor footprints, Nature, 2012.

[32] Boyle et al., High-resolution genome-wide in vivo footprinting of diverse transcription factors in human cells, Genome Res., 2011.

[33] Sung et al., DNase footprint signatures are dictated by factor dynamics and DNA sequence, Mol Cell, 2014.

[34] Piper et al., Wellington: a novel method for the accurate identification of digital genomic footprints from DNase-seq data, Nucleic Acids Res., 2013.

[35] Gusmao et al., Analysis of computational footprinting methods for DNase sequencing experiments, Nature methods, 2016.

[36] Ramachandran et al., BIDCHIPS: bias decomposition and removal from CHIP-seq data clarifies true binding signal and its functional correlates, Epigenetics Chromatin, 2015.

[37] Koohy et al., A Comparison of Peak Callers Used for DNase-Seq Data, PLoS One, 2014.

[38] Tanay A., Extensive low-affinity transcriptional interactions in the yeast genome, Genome Res. 2006.

[39] Crocker et al., Low affinity binding site clusters confer hox specificity and regulatory robustness, Cell, 2015.

[40] Roider et al., PASTAA: identifying transcription factors associated with sets of co-regulated genes, Bioinformatics, 2009.

[41] Thomas-Chollier et al., Transcription factor binding predictions using TRAP for the analysis of ChIP-seq data and regulatory SNPs, Nat. Protoc., 2011.

[42] Costa et al., Predicting gene expression in T cell differentiation from histone modifications and transcription factor binding affinities by linear mixture models, BMC Bioinformatics, 2011.

[43] van Bömmel. Prediction of transcription factor co-occurrence using rank based statistics, PhD thesis, Freie Universität Berlin, 2015.

[44] Keilwagen et al., Varying levels of complexity in transcription factor binding motifs, Nucleic Acids Res., 2015.

[45] Alipanahi et al., Predicting the sequence specificities of DNA- and RNA-binding proteins by deep learning, Nature Biotechnology, 2015.

[46] Jayaram et al., Evaluating tools for transcription factor binding site prediction, BMC Bioinformatics, 2016.

[47] Turatsinze et al., Using RSAT to scan genome sequences for transcription factor binding sites and cis-regulatory modules, Nat. Protoc., 2008.

[48] Frith et al., Detection of functional DNA motifs via statistical over-representation, Nucleic Acids Res., 2004.

[49] Beckstette et al., Fast index based algorithms and software for matching position specific scoring matrices, BMC Bioinformatics, 2006.

[50] Li et al., Regression Analysis of Combined Gene Expression Regulation in Acute Myeloid Leukemia, PLoS Computational Biology, 2014.

[51] Wilczynski et al., Predicting Spatial and Temporal Gene Expression Using an Integrative Model of Transcription Factor Occupancy and Chromatin State, PLoS Computational Biology, 2012.

[52] Ferdous et al., Predicting gene expression from genome wide protein binding profiles, ScienceDirect, 2017.

[53] Wu et al., Identifying differential transcription factor binding in ChIP-seq, Front Genet., 2015.

[54] Cheng et al., Understanding transcriptional regulation by integrative analysis of transcription factor binding data, Genome Res., 2012.

[55] Chen et al., Differential chromatin profiles partially determine transcription factor binding, PLoS One, 2017.

[56] Heinig et al., histoneHMM: Differential analysis of histone modifications with broad genomic footprints, BMC Bioinformatics, 2015.

[57] Shao et al., MAnorm: a robust model for quantitative comparison of ChIP-Seq data sets, Genome Biology, 2012.

[58] Xiao et al., Comparative epigenomics: defining and utizling epigenomic variations across species, time-course and individuals, Wiley Interdiscip. Rev. Syst. Biol. Med. ,2014.

[59] Rendeiro et al., Chromatin accessibility maps of chronic lymphocytic leukaemia identify subtype-specifc epigenome signatures and transcription regulatory networks, Nature Communications, 2016.
